# Occupational Stress, Burnout, and Quality of Life in Radiographers: A Scoping Review of Workforce Well-Being

**DOI:** 10.3390/healthcare14040538

**Published:** 2026-02-22

**Authors:** Pedro Ramalho, António Nunes, Fernanda M. Silva, André Ramalho, Gonçalo Flores, Beatriz Santos, Ricardo Ferraz, Henrique Neiva, Pedro Duarte-Mendes

**Affiliations:** 1 Department of Management and Economics, University of Beira Interior (UBI), 6201-001 Covilhã, Portugal; ramalho.pedro@sapo.pt (P.R.); anunes@ubi.pt (A.N.); 2 ULS Castelo Branco, Amato Lusitano Hospital, 6000-085 Castelo Branco, Portugal; 3 NECE, Research Center for Business Sciences, 6200-209 Covilhã, Portugal; 4 School of Education and Communication, University of Algarve, 8005-139 Faro, Portugal; fmadsilva@ualg.pt; 5 CIPER, Faculty of Sport Sciences and Physical Education, University of Coimbra, 3004-531 Coimbra, Portugal; 6 Sport Physical Activity and Health Research and Innovation Center, SPRINT, 6000-216 Castelo Branco, Portugal; andre.ramalho@ipcb.pt; 7 Department of Sports and Well Being, Polytechnic Institute of Castelo Branco, 6000-084 Castelo Branco, Portugal; 8 Faculty of Sport, University of Porto, 4099-002 Porto, Portugal; goncalofloresft@outlook.com; 9 ESECS, Polytechnic University of Leiria, 2411-901 Leiria, Portugal; beatrizfilipa97@gmail.com; 10 Department of Sport Sciences, University of Beira Interior, 6201-001 Covilhã, Portugal; ricardompferraz@gmail.com (R.F.); hpn@ubi.pt (H.N.); 11 Research Center in Sports Sciences, Health Sciences, and Human Development (CIDESD), 6201-001 Covilhã, Portugal

**Keywords:** radiologic technologists, occupational stress, emotional exhaustion, well-being, psychosocial risk

## Abstract

**Background/Objectives**: We conducted a scoping review to map peer-reviewed evidence on occupational stress, burnout, and quality of life among radiographers and radiologic technologists and to identify measurement tools and reported consequences. **Methods**: Searches were conducted in Web of Science, Scopus, and PubMed. Eligible studies enrolled radiographers/radiologic technologists who were healthy adults; assessed at least one target construct (occupational stress, burnout, or quality of life) using validated instruments; and used cross-sectional, experimental, quasi-experimental, longitudinal, or mixed-methods designs. Articles published from 1995 onward in English, French, Spanish, or Portuguese were considered. Two reviewers independently screened, extracted data, and appraised methodological quality using Quality Assessment with Diverse Studies (QuADS). The synthesis was narrative only. **Results**: Of 2701 records, 10 studies from nine countries met inclusion. Most were cross-sectional, and two used mixed methods. Sample sizes ranged from 38 to 864. Frequently used instruments included MBI-HSS, OSI-R, HSE Indicator Tool, and SOC-13. Across studies, radiographers reported high stress and burnout—particularly emotional exhaustion and depersonalization—alongside reduced quality of life in multiple domains. Recurrent stressors involved workload and staffing pressures, role demands, anxiety about radiation exposure, and limited recognition. These factors were associated with intention to leave and a lower sense of coherence. **Conclusions**: The evidence base is largely cross-sectional, uses heterogeneous measures, and often relies on modest samples, with overall methodological quality mostly moderate. Findings indicate a persistent psychosocial risk profile in radiography and underscore the need for organizational and managerial actions—such as workplace physical activity programs—to reduce stress and burnout and protect the quality of life in this workforce.

## 1. Introduction

Healthcare professionals are exposed to multiple occupational stressors that can compromise both their well-being and the quality of patient care. Among these professionals, radiographers and radiologic technologists are particularly vulnerable due to the technical complexity of their tasks, exposure to ionizing radiation, high workloads, and the constant need for precision and patient interaction. Over the past two decades, increasing evidence has shown that these conditions may contribute to occupational stress and burnout, phenomena that have profound implications for health systems worldwide [1,2,3].

The working environment of radiographers is frequently characterized by high patient throughput, staff shortages, and shift-based work schedules, including night and weekend duties. These professionals often operate across multiple clinical settings, such as emergency departments, operating rooms, and outpatient services, which increases task variability and cognitive and emotional demands [1,2,3]. In addition, the physical requirements of radiographic practice (including prolonged standing, repetitive movements, and awkward postures) contribute to fatigue and musculoskeletal complaints, reinforcing the link between physical strain and psychological distress [2].

Burnout, recognized by the World Health Organization as an occupational phenomenon rather than a medical disorder, is characterized by emotional exhaustion, depersonalization, and reduced personal accomplishment [4]. It is associated with decreased job satisfaction, reduced productivity, and higher turnover intentions. In radiography, the literature suggests that burnout may be a relevant occupational health concern, but findings are dispersed across contexts and assessment approaches [1,2,3]. However, the available evidence remains fragmented and heterogeneous, with differences in methodological quality, sample characteristics, and assessment instruments. These gaps hinder the formulation of comprehensive occupational health strategies targeted at this professional group.

Controversy persists regarding the factors that most strongly predict stress and burnout in radiography. While some studies emphasize individual variables—such as gender, age, and coping style—others highlight organizational aspects including workload, shift work, and leadership quality. Moreover, the recent literature has highlighted interest in potential protective factors (e.g., self-compassion, coping strategies, and workplace physical activity), in buffering psychological distress. However, evidence remains heterogeneous and not yet consolidated [1,2,3]. Despite this growing interest, no consensus has been reached on the most effective interventions or on the standardized tools to assess occupational stress, burnout, and quality of life in radiographers.

In this context, the present scoping review aims to systematically map and synthesize peer-reviewed evidence on occupational stress, burnout, and quality of life among radiographers and radiologic technologists. Specifically, this review aims to describe the levels of stress, burnout, and quality of life; identify the assessment instruments used to measure these variables, summarize the main risk factors and individual and organizational consequences reported; and examine the potential mitigation strategies proposed in the literature. For clarity, study-level findings from eligible articles are presented exclusively in the Results and interpreted in the Discussion.

## 2. Materials and Methods

### 2.1. Study Design

This scoping review was conducted in accordance with the PRISMA-ScR (Preferred Reporting Items for Systematic Reviews and Meta-Analyses extension for Scoping Reviews) guidelines [5], with a protocol registered in the Open Science Framework (10.17605/OSF.IO/2RCUF), which provides specific standards for the development of this type of study. In line with these guidelines, the review followed the fundamental stages of the process, namely: the formulation of the research question, the definition of eligibility criteria, the identification of information sources, the selection of studies, the extraction and organization of data, and finally, the analysis, synthesis, and presentation of the results.

### 2.2. Eligibility Criteria: Inclusion and Exclusion

The eligibility criteria were defined in advance, in accordance with the objective of this scoping review and the PRISMA recommendations, in order to ensure consistency and transparency in the study selection process. The PCC framework (Population, Concept, Context) was used to ensure clarity and methodological consistency. Eligible studies had to be peer-reviewed, involve radiographers (Population), assess work-related stress, burnout, or quality of life (Concept), and occur in any healthcare setting (Context). Inclusion criteria: studies with samples composed of radiographers; participants being healthy individuals; assessment of at least one of the variables of interest (stress and/or burnout and/or quality of life) using validated scales; cross-sectional, experimental, quasi-experimental, longitudinal, or mixed-methods studies; and articles published since 1995 in English, French, Spanish, or Portuguese. Exclusion criteria: reviews, protocols, combined assessments, opinion articles, editorials, letters to the editor, conference abstracts, or non-scientific reports, COVID-19 studies, artificial intelligence studies; duplicate studies or those whose data were not available in full text; and investigations that, although initially identified, did not address the research question upon full-text reading.

### 2.3. Search Strategy

Initially, the electronic databases considered most appropriate for the scientific field in question were selected, namely Web of Science, SCOPUS, and PubMed, with access until March 2025. For each database, a set of search terms was defined, comprising controlled descriptors (e.g., MeSH) and free-text terms related to the review’s central concepts. These terms were combined using Boolean operators (“AND”, “OR”), and, whenever necessary, quotation marks were used to increase the sensitivity and specificity of the search.

Predefined limits were also applied in accordance with the eligibility criteria, namely articles in Portuguese, English, Spanish, and French published since 1995. In addition to the electronic search, a manual search of the reference lists of the included articles was carried out to identify additional relevant studies that had not been retrieved initially (snowballing).

The set of controlled descriptors, selected according to the specific vocabularies of each database, together with keywords and appropriate use of Boolean operators, enabled the construction of a comprehensive and precise search strategy. Thus, the combination of these elements resulted in the following search strategy, used to identify the studies relevant to the topic under analysis:

((radiographer OR “radiology technologist” OR “radiologist technician” OR radiologist OR radiotherapist OR “radiologic technologist” OR “radiation oncologists” OR “radiation therapist” OR “attending radiologist” OR” interventional radiologist” OR “radiology residents” OR “radiology fellow*” OR “radiology attending” OR “radiology consultant”) AND (exercise* OR “physical exercise” OR “physical activity” OR training OR “combined training” OR “aerobic exercises” OR strength OR program* OR mobility OR stretch) AND (burnout OR “burned out” OR “professional burnout” OR “occupational burnout” OR “career burnout” OR “occupational stress” OR “work stress” OR “professional stress” OR “workplace stress” OR “work-related stress” OR “job stress” OR “health related quality of life” OR “quality of life” OR HRQOL OR “life quality”))

### 2.4. Study Selection and Data Extraction

The study selection process was carried out in several stages, following the PRISMA guidelines [6]. In the first stage, all results obtained from the different databases were exported to bibliographic management software (EndNote X9), where duplicate records were automatically removed and subsequently eliminated manually. Next, titles and abstracts were independently screened by two reviewers, based on the previously defined inclusion and exclusion criteria. Potentially eligible articles were retrieved in full text and analyzed in detail to confirm their relevance. In cases of disagreement between reviewers, the final decision was reached by consensus or, when necessary, with the involvement of a third reviewer.

Data extraction was performed systematically using a pre-developed extraction form. This form included the main variables of interest, such as authorship, year, country, study design, sample details, instruments, and results. Whenever relevant, additional information pertinent to addressing the research questions was also recorded.

All extracted data were organized into tables and subsequently synthesized according to the review objectives, to enable descriptive analysis and the identification of patterns, trends, and gaps in the literature.

### 2.5. Quality Assessment

The Quality Assessment with Diverse Studies (QuADS) tool, developed by Harrison et al. [7], was specifically designed to assess the methodological quality of studies included in systematic reviews that encompass mixed-methods, multiple-methods, or diverse methodological approaches. Unlike tools intended exclusively for quantitative or experimental studies—such as randomized controlled trials or observational studies—QuADS was developed to address the methodological heterogeneity often found in reviews that include qualitative, non-experimental quantitative, or mixed-methods studies.

QuADS enables reviewers to consider, in an integrated manner, different dimensions of methodological quality, such as the clarity of objectives, theoretical justification, participant involvement, author reflexivity, and the practical relevance of findings. This tool does not establish rigid cut-off points, as its authors recognize that any standardized classification attempt could be arbitrary. Instead, it promotes a reflective approach, allowing for the interpretation of criteria to be adapted to the specific context and objectives of the review.

The instrument consists of 13 items that analyze aspects related to the clarity of objectives, adequate design, theoretical justification, description of context, stakeholders involved, rigorous data collection, among others. Each item is assigned a score from 0 to 3, allowing a detailed assessment of the quality of each study. The final score has a maximum of 39 points.

The assessment was carried out independently by two reviewers. In cases of disagreement, discrepancies were resolved by consensus or, when necessary, with the involvement of a third reviewer. The quality assessment results were recorded systematically and subsequently presented in tabular form, providing a comparative overview of the methodological quality of the included studies and facilitating the interpretation of the findings of this scoping review.

## 3. Results

### 3.1. Study Identification and Selection

**Figure 1:**
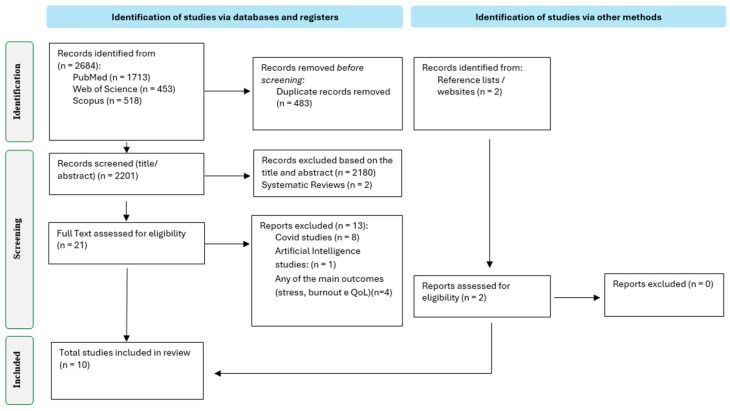
PRISMA flow diagram for the selection of studies.

The flowchart presented in Figure 1 provides an insightful overview of the results of the bibliographic search. During the scoping review, a total of 2699 records were identified through database searches, and two additional studies were identified through reference list screening. After duplicate removal, the number was reduced to 2201 studies. From this group, a careful screening process excluded 2178 articles with irrelevant titles and abstracts, leaving 23 full-text articles for further analysis. Finally, 15 of these articles were excluded after full-text reading; the reasons for their exclusion are clearly explained in Figure 1. In the end, 10 studies were selected that met the rigorous inclusion criteria and provided a compelling justification for their inclusion in this review. This rigorous selection process ensured that only studies relevant and consistent with the research objectives were included.

### 3.2. Study Characteristics

Table 1 provides an overview of the main characteristics of the 10 studies included in the analysis. The studies were conducted in various countries—Iran, China, France, Ireland, Saudi Arabia, Jordan, Portugal, Malaysia, and the United Kingdom. Eight of the studies employed quantitative approaches [8,9,10,11,12,13,14,15], using validated questionnaires to measure levels of stress, burnout, or well-being. Two studies adopted mixed-methods approaches [16,17], incorporating qualitative dimensions such as open-ended comments and descriptive analyses of perceptions. Sample sizes ranged from 38 to 864 participants, most of whom were active radiographers, with average ages of 30 to 40 years. Most studies focused on healthy adults living in the community. Data collection methods primarily involved validated questionnaires.

### 3.3. Study Methodology

All 10 articles analyzed adopted cross-sectional methodologies, consisting of observational studies with data collected at a single point in time. Although the search timeframe was broad, only ten studies met eligibility, indicating limited eligible evidence in the peer-reviewed literature. While this methodological choice limits the ability to establish causal relationships, it is widely used in studies assessing the prevalence of psychological and behavioral phenomena.

Eight of the studies employed quantitative approaches [8,9,10,11,12,13,14,15], using validated questionnaires to measure levels of stress, burnout, or well-being. Two studies adopted mixed methods approaches [16,17], incorporating qualitative dimensions such as open-ended comments and descriptive analyses of perceptions.

Regarding the origin of the samples, the studies differed significantly in terms of institutional context: some were conducted in public hospitals (as in Portugal and Iran), others in mixed environments (as in Jordan), and some included multiple countries (as in the international study by Kennedy et al., [8]. This diversity allows for a comparative interpretation of the influence of cultural variables, labor policies, and healthcare systems on the experience of stress.

### 3.4. Sample Characteristics

The sample size of the studies analyzed varies considerably, reflecting the heterogeneity of the contexts in which they were conducted. The largest study, conducted by Shubayr and Alashban [17], involved 864 radiographers in Saudi Arabia, while the smallest, by Videira and Ventura [14], included only 38 participants from a Portuguese hospital. Between these extremes, most studies reported samples ranging from 43 to 253 participants, the majority consisting of active professionals, with an average age between 30 and 40 years.

Regarding gender distribution, most samples show a significantly higher proportion of women, except for studies conducted in Saudi Arabia [17] and Jordan [11], where men were the majority. This finding may reflect distinct cultural and institutional contexts. In the United Kingdom, for example, Rutter and Lovegrove [15] studied exclusively women, as mammography is performed solely by female professionals. The Portuguese studies provided greater detail on working conditions, highlighting differences between public and private services and between small and large teams, thereby enriching the contextual understanding of risk factors.

### 3.5. Assessment Instruments

The analysis of the ten studies revealed a diversity of instruments used to assess different dimensions of occupational stress, burnout, and quality of life. This methodological variety, while enriching, also represents a limitation to the direct comparison of results across different contexts.

To measure occupational stress, several internationally validated instruments were applied. The most frequently used was the Occupational Stress Inventory–Revised (OSI-R), which was employed in studies conducted in Iran [13] and Jordan [11]. This tool assesses dimensions such as workload, professional roles, interpersonal relationships, and individual coping resources. Kennedy et al. [8] used the HSE Management Standards Indicator Tool, developed in the United Kingdom and widely adopted in occupational health research, which identifies psychosocial risk factors related to demands, control, support, relationships, role, and organizational change. Other studies used structured sociodemographic and occupational questionnaires, such as those by Shubayr & Alashban [17], which included specific questions on experiences of bullying and perceptions of institutional support, and by Videira Ventura [14], which incorporated indicators of workload and technical versatility.

Regarding burnout syndrome, the reference instrument was the Maslach Burnout Inventory–Human Services Survey (MBI-HSS), applied in four studies [8,9,10,14]. The MBI-HSS assesses the three classical dimensions of the syndrome—emotional exhaustion, depersonalization, and personal accomplishment—providing consistent data on the prevalence of clinically relevant burnout levels among radiographers. In some cases, such as Cui et al. [12], burnout was also analyzed in relation to psychological variables, such as the sense of coherence, using complementary scales, thereby broadening understanding of the phenomenon from a more integrative perspective.

### 3.6. Methodological Quality of Studies Included

The methodological quality assessment is presented in Table 2. The majority of studies were rated as having moderate quality. In several cases, the studies demonstrated well-organized structures and adequate theoretical grounding but lacked reflexivity and offered only brief discussions of limitations, thereby weakening the research’s critical depth. Some works displayed solid methodological rigor and sound practical applications, placing them in the moderate-to-high quality range, though they remained limited by insufficient participant engagement and reflective depth. Others featured clear and consistent analyses but failed to advance methodological critique or acknowledge inherent weaknesses in their approaches. Conversely, specific articles exhibited moderate-to-low quality, either due to restricted instruments, superficial analyses, or limited reflexivity, compromising the robustness of their findings. A few studies also had small samples or shallow analytical approaches, constraining the validity and generalizability of their results. Overall, the articles were well structured and theoretically supported, though they shared weaknesses related to the absence of critical reflexivity, limited discussion of methodological limitations, and, in some cases, insufficient methodological robustness.

## 4. Discussion

The present review aimed to understand the levels of occupational stress, burnout, and quality of life among radiographers, identify the assessment instruments used and the main associated factors, analyze their individual and organizational consequences, and explore the potential mitigation strategies proposed in the studies. Ten national and international studies were comparatively analyzed, covering contexts such as Iran, China, France, Ireland, Saudi Arabia, Jordan, Portugal, Malaysia, and the United Kingdom. Despite methodological diversity, all converge in revealing that radiographers experience high levels of psychological, emotional, and physical exhaustion, confirming the profession’s particular vulnerability to occupational stress and burnout [8,9,10].

Across studies, common risk factors include work overload, shift work, multiple role accumulation, low recognition, poor management, and lack of organizational support [8,9,16]. Contractual instability, such as fixed-term contracts, and workplace bullying—reported by up to 89% of professionals in certain contexts [17]—further intensify strain. These findings align with Rutter and Lovegrove’s [15] observation that role ambiguity and insufficient recognition contribute to dissatisfaction and intention to leave the profession. Physical consequences, such as musculoskeletal complaints affecting the lower back, neck, and shoulders [11], reinforce the link between emotional exhaustion and somatic symptoms.

Sociodemographic variables also influence these outcomes. Female professionals consistently emerge as more vulnerable to burnout and stress, possibly reflecting the double burden of professional and family responsibilities and persistent inequalities in career advancement. The role of age and length of service appears ambivalent: while greater experience may enhance coping skills and emotional resilience [16], prolonged exposure to demanding environments can intensify exhaustion [9].

Some studies introduced moderating and protective factors. Self-compassion emerged as a key variable, associated with greater emotional resilience and lower burnout [16]. Positive coping strategies, such as regular physical activity and leisure pursuits, were also identified as protective mechanisms [10,14]. However, despite these insights, few studies offered empirically tested interventions to mitigate occupational stress, emphasizing instead theoretical recommendations for institutional support and recognition of radiographers within multidisciplinary teams.

Within this context, the analysis highlights the importance of promoting physical activity and workplace exercise as preventive strategies. Several studies documented significant associations between occupational stress and physical complaints, suggesting that regular exercise can reduce injuries linked to repetitive effort and prolonged postures while enhancing overall well-being. The relationship between physical and emotional health is evident: exercise fosters emotional balance, reduces anxiety, and mitigates depersonalization associated with burnout [18,19].

Empirical evidence from intervention studies supports this perspective. In a controlled study [20], a workplace exercise program led to significant improvements in quality of life—particularly in the physical (*p* = 0.046) and environmental (*p* = 0.032) domains—and reductions in perceived stress (*p* = 0.013) and burnout (*p* < 0.05) among radiographers in the experimental group. In contrast, the control group showed no significant changes. The intervention promoted psychophysiological recovery, enhanced perceptions of institutional support, and strengthened emotional regulation. These effects are consistent with research linking regular physical activity to improved energy, mood, and concentration, as well as lower burnout levels [8,16].

Overall, the ten studies confirm the multifactorial nature of occupational stress among radiographers, influenced by both personal and organizational variables. They also demonstrate that structured, low-cost interventions—such as workplace exercise programs—can effectively promote physical and psychological well-being, reduce stress and burnout, and improve quality of life. For policymakers and healthcare institutions, the findings underscore the urgency of implementing comprehensive occupational health policies that combine psychological support, fair working conditions, and initiatives to foster emotional resilience and physical activity in the workplace. Such integrated strategies are essential to ensure sustainable professional practice, enhance job satisfaction, and ultimately improve the quality and safety of patient care [8,9,10,14,16,18,19,20].

### Study Limitations

The present analysis of the ten studies has several limitations that should be acknowledged. First, most investigations adopted a cross-sectional design, which prevents the establishment of causal relationships among the variables studied. Although associations can be identified between factors such as gender, years of service, or working conditions and levels of stress or burnout, it is not possible to determine the direction of these relationships or to confirm whether stress results directly from these factors or whether the phenomena are bidirectional in nature.

Another relevant limitation concerns methodological heterogeneity. The studies employed a variety of assessment instruments, including the MBI-HSS, OSI-R, HSE Indicator Tool, IPWBW, and structured questionnaires, which complicates direct comparison of results and hinders the development of more robust quantitative syntheses. In addition, some studies presented only categorized data by age groups or professional categories, without providing means or standard deviations, thereby limiting the precision of comparisons.

The geographical diversity of the studies, while representing a strength in terms of scope, also introduces constraints, as working conditions, cultural factors, and health policies vary substantially across countries such as Portugal, Iran, France, Saudi Arabia, and Malaysia. This factor makes generalizing the findings risky, since the impact of occupational stress may differ across specific organizational and sociocultural contexts.

Furthermore, there was considerable variation in sample sizes, ranging from studies with fewer than 50 participants to those with several hundred participants. This disparity compromises the representativeness of some conclusions, particularly in smaller studies, where the likelihood of sampling bias is higher.

Another limiting aspect relates to the scarce exploration of mediating and contextual variables. Although well-established risk factors such as workload, shift work, bullying, and lack of institutional support were identified, few investigations examined in depth the more subjective dimensions of stress—such as perceived recognition, organizational culture, or leadership style—leaving gaps in the understanding of subtler determinants of occupational stress.

The limited number of included studies may constrain the comprehensiveness of conclusions, but it also maps an evidence gap consistent with the purpose of a scoping review.

Finally, it is important to note that most studies did not include empirically tested intervention proposals, relying instead on theoretical or descriptive recommendations. This lack of applied research reduces the immediate practical contribution of the existing literature to the evidence-based implementation of occupational health policies.

## 5. Conclusions

The analysis of the studies included in this synthesis shows that radiology technicians report levels of occupational stress and burnout, associated with a combination of individual, organizational and contextual factors related to different health profession contexts. The available evidence thus suggests the demanding nature of the working conditions of these professionals and the potential negative implications for their well-being. Intervention strategies focused on professional recognition, psychological support, and the promotion of physical activity appear to play an important role in preventing burnout and improving overall quality of life. Therefore, it is essential that healthcare institutions adopt sustainable policies that foster balanced, healthy, and emotionally safe work environments.

The review also identified substantial heterogeneity in the instruments used to assess these constructs. Although validated tools such as the Maslach Burnout Inventory–Human Services Survey (MBI-HSS), the Occupational Stress Inventory–Revised (OSI-R), and the HSE Management Standards Indicator Tool were most commonly applied, the lack of standardization limits comparability across studies and highlights the need for more harmonized assessment approaches in future research.

Across the included studies, the main risk factors associated with stress and burnout were predominantly organizational and included high workload, staff shortages, shift work, role ambiguity, workplace bullying, limited professional recognition, and insufficient managerial support. These factors were consistently linked to important individual and organizational consequences, such as reduced job satisfaction, physical and psychological health complaints, increased intention to leave the profession, and potential risks to workforce sustainability and quality of care.

Although the overall methodological quality of the evidence was mostly moderate and largely based on cross-sectional designs, several studies highlighted potential mitigation strategies. Protective factors such as self-compassion, effective coping strategies, and workplace physical activity were identified as promising approaches to reducing stress and burnout and enhancing well-being. However, empirically tested interventions remain scarce, underscoring the need for future longitudinal and intervention-based studies.

In summary, promoting the physical and psychological well-being of radiographers should be regarded as an institutional priority and an essential condition for delivering high-quality, safe, and compassionate healthcare.

Taken together, the findings of this scoping review support the conclusion that radiographers and radiologic technologists constitute a group particularly affected by work-related stress. Across diverse healthcare settings and countries, the included studies consistently reported elevated levels of occupational stress and burnout, especially emotional exhaustion, alongside negative impacts on quality of life. This vulnerability appears to be closely linked to the specific characteristics of the radiography working environment, which combines high technical demands, responsibility for radiation safety, physical strain, shift work, and frequent exposure to organizational stressors such as workload pressure, staff shortages, limited recognition, and insufficient managerial support. The convergence of these factors, repeatedly observed across studies, provides a coherent basis for interpreting radiographers as a professional group at increased psychosocial risk when compared with broader healthcare populations.

## Figures and Tables

**Table 1:** Characteristics of Studies.

Author, Year, and Country	Study Design	Sample Size (*n* Total; *n* ♂/*n* ♀; Age)	Assessment Instruments	Main Outcomes	Main Results	Main Conclusions
Cui et al., 2022, [12] China	Cross-sectional study	277 (149 ♂/128 ♀) Age (years old) 20–29: 158 (57%) 30–39: 78 (28.2%) >40: 41 (14.8%) predominantly in age 20 to 39 (85.2%)	SOC-13; MBI-HSS	Burnout; Sense of Coherence (SOC); Radiation anxiety; Job leaving intention	Mean SOC-13 was 50 (SD = 13.5); burnout: 79.7 (SD = 25.7). There is a significant linear correlation between SOC and burnout. Anxiety about radiation exposure is significantly associated with the intention to leave the job. Participants without anxiety have higher SOC and lower burnout. Participants with no intention of leaving the job also have better SOC and lower burnout. Anxiety and intention to leave the job: 28.5% of participants expressed a desire to leave the profession. 48.4% were undecided. Moderate/severe anxiety is associated with a greater intention to leave. Most reported reasons for possible abandonment: Low income (70.2%); Anxiety about the effects of radiation on personal health (69.7%); Effects on children’s health (57.6%); Excessive workload (47%).	High burnout and low SOC; radiation anxiety increases job-leaving intention; stress education programs are needed about radiation protection
Kennedy et al., 2025, [8] Ireland and United Kingdom	Cross-sectional survey	245 (207 ♀/33 ♂) The largest age group was 25 to 34 (*n* = 76.31%)	MBI; Job Satisfaction Survey (JSS)	Burnout; Job satisfaction	25% of radiologists experience high burnout in at least one dimension. 11.4% experience high burnout in all dimensions. Depersonalization was the dimension with the highest burnout (48.6%). Job satisfaction: 44.2% dissatisfied; 43.7% ambivalent; 12.1% satisfied. Greatest dissatisfaction: salary (77.1%), rewards (76.4%), benefits (73.5%), promotions (68%). Greatest satisfaction: nature of work (66.4%), colleagues (40.8%). Main burnout factors: workload, lack of recognition, time pressure. Factors that improve satisfaction: good relationships with colleagues, patient interaction, and autonomy. Statistical relationships: Age 25–34 had a higher SD than 45–54. Lower personal fulfillment in younger people (18–34 years old). Workload correlated with emotional exhaustion	Depersonalization with moderate to high burnout; dissatisfaction driven by pay and recognition; HR support needed. Investments in human capital, empathetic leadership, and collaborative environments are recommended to increase retention and efficiency of healthcare services.
Shubayr & Alashban, 2024, [17] Saudi Arabia	Mixed-method cross-sectional study (quantitative and qualitative)	864 (642 ♂/222 ♀) The age profile was skewed towards younger adults with 45% (*n* = 390) aged between 25 and 34 years and 41% (*n* = 357) aged 35–44 years	HSE-IT questionnaire	Organizational stressors; Psychosocial indicators	High stress in demands and management support; 89% reported bullying. Greater stress in women. Greater stress in those who work at night. Main causes of stress (qualitative): Unsupportive management, excessive workload, lack of recognition and feedback, personal harassment, and role ambiguity	Urgent need for institutional interventions and stress management policies. Suggestions for reducing stress include: Increased staffing. Better management and regular feedback. Developing teamwork. Better communication and supportive policies. Regular training and appropriate breaks
Che Abdullah et al., 2023, [10] Malaysia	Quantitative cross-sectional survey	49 (22 ♂/27 ♀) Range: 20–59 years	Adapted Ashong et al. questionnaire	Stress levels; Contributing factors; Coping strategies	73% reported moderate stress; key stressors: lack of support from supervisors, relationships with colleagues, physical working conditions, and physical and mental workload	Moderate stress is common; physical activity is used as coping; stress management training is recommended
Jacquet et al., 2024, [16] France	Exploratory mixed-method study	253 (213 ♀/40 ♂) 32.9 ± 10.8 (range: 20–63 years)	IPWBW; Self-Compassion Scale	Psychological well-being at work: Self-compassion and Sociodemographic factors	Moderate self-compassion; low well-being; factors such as age, gender, and experience influenced well-being levels. Participants with higher self-compassion reported less emotional exhaustion.	Self-compassion and psychological well-being are low. Well-being training is recommended. Lack of recognition and weak interpersonal relationships are critical factors. Self-compassion has been shown to be a protective factor against burnout, emotional exhaustion, and stress. Younger people and women are more vulnerable. Increase the visibility and recognition of the profession. Promote self-compassion training. Invest in mindfulness and emotional training programs. Develop protocols for cooperation and mutual support among colleagues. Self-compassion should be considered an occupational well-being tool.
Videira & Ventura, 2008, [14] Portugal	Quantitative cross-sectional study	38 (10 ♂/28 ♀) only refer to females, aged between 30 and 39 years old.	Stress-Inducing Agents in Healthcare Professionals (HPSIAS); Evoked Response Scale (ERE)	Stress agents; Symptoms; Focus on working hours and diversity of functions	High stress in the operating room and Computed Tomography; Professionals who work in multiple areas have higher levels. 1. Area with the most stress: the operating room was the most stressful (average 3.06/5), the ward (2.89), and the emergency room (2.39). 2. Stress-inducing factors (HPSIAS): (a) Working conditions: Too much work in a short time was the most reported (2.61/4). Exposure to biological and infectious risks, lack of human and material resources; (b) Job performance: Feelings of underutilization of skills were the most notable (2.21); fear of making mistakes and lack of preparation; (c) Interpersonal relationships: Role conflict was the most stressful (2.00); Difficulties in relationships with colleagues and superiors; (d) Changes at work stood out as the main source of stress (2.16); (e) Difficult work situations: Lack of institutional support was the highest factor in the study (2.63), followed by the absence of support from superiors and colleagues; (f) Perception of stress: The SU was the area where technicians most perceived work as a source of stress (average: 2.42). 3. Evoked Responses (ERE): (I) Physiological symptoms: Permanent fatigue was the most common (1.58/4), sleep disturbances, headaches, back pain, gastric upset; (II) Psychological symptoms: Irritation and anger were the most evident (1.87); forgetfulness, difficulty concentrating, dissatisfaction, negative attitudes; (III) Behavioral symptoms: Need to eat more often; loss of interest in physical exercise (both 1.29). 4. Stress-reduction mechanisms: 13.2% increased tobacco consumption, 5.3% increased tranquilizer consumption, no increase in alcohol consumption, most cases occur in emergency departments, and technicians working in multiple areas. 5. Absenteeism and accidents: Technicians working in multiple areas reported workplace accidents; Average annual absences: 13.65 days. 6. Career stress progression: 55.3% reported increased stress throughout their career; Emergency departments and multiple areas technicians felt the greatest increase in stress. 7. Job satisfaction: 47.4% are satisfied; Emergency departments and multiple areas technicians were the most dissatisfied. 8. Current career choice: 71.1% would choose the profession again; the Emergency department was the only area where the majority did not choose it again	Stress is prevalent among multi-role technicians; training and institutional support are advised. redistribution of tasks
Vieira da Silva & Pereira, 2020, [9] Portugal	Quantitative multicenter cross-sectional study	122 (47 ♂/75 ♀) 38.3 (±9.3)	MBI-HSS; Demographic questionnaire	Burnout (Emotional Exhaustion, Depersonalization, Reduced Personal accomplishment); Sociodemographic associations (gender and length of service) impact of work on self-esteem, family, and social life	39.3% had high EE; 29.5% depersonalization. 48.4% low reduced personal accomplishment Higher burnout in women and mid-career professionals	Burnout is common; emotional exhaustion is linked to gender, workload, and support needs
Rutter & Lovegrove, 1995, [15] UK	Postal survey (cross-sectional)	103 (all ♀) 40–44 years	Postal Questionnaire: Malaise Inventory; Role conflict/ambiguity scales; Satisfaction scale	Stress; Job satisfaction	30% had high stress; role ambiguity predicted job dissatisfaction. Main causes: lack of communication and work-family conflict. Difficulty in reconciling schedules. Only 17% are very satisfied with their work, more than 50% are considering changing jobs, 12% thought it was very likely or extremely likely that they would look for a new job in the following year. Role ambiguity was what contributed to dissatisfaction. Stress is not related to age, marital status, position, or time at work. The intention to leave work is associated with dissatisfaction, not stress.	Technicians have high levels of stress and low satisfaction. Clarifying job roles and improving communication may enhance job satisfaction
Alhasan et al., 2014, [11] Jordan	Quantitative cross-sectional study (3 hospitals)	74 (40 ♂/34 ♀) 30 ± 3.3 to 33.6 ± 1.4	Custom questionnaire (WRS, MSD, symptoms)	Occupational stress; Musculoskeletal complaints; Stress symptoms	Public hospitals had the highest stress and MSD; stress correlated with MSD symptoms	Ergonomic and psychological interventions are needed in public hospitals
Masoumbeigi et al., 2024, [13] Iran	Cross-sectional study with a questionnaire	43 (12 ♂/31 ♀) 31.16 ± 8.48	Osipow Stress Inventory—Revised (OSI-R)	Stress levels; Demographic factors; Working hours; Contract type and salary	Higher stress in women, fixed-term contracts, and higher income; fewer working hours are associated with higher stress	Stress is strongly linked to gender, contract type, and hours; institutional programs are recommended

♂ male; ♀ female.

**Table 2:** Quality Assessment of Studies.

	Clear Objectives	Appropriate Design	Theoretical Justification	Context Description	Stakeholders Involved	Rigorous Data Collection	Rigorous Data Analysis	Reflexivity	Discussion of Limitations	Scientific Contribution	Clarity of Results	Consistency of Conclusions	Practical Relevance	Total Score	Observations
Article 1	3	2	2	2	1	3	2	0	1	2	3	2	2	25	Moderate-to-low quality. Lacks reflexivity and discussion of limitations.
Article 2	3	2	3	2	1	3	2	0	2	3	3	2	2	28	Moderate quality. Well-structured, but lacks participant involvement.
Article 3	3	2	3	2	1	3	2	0	2	3	3	2	3	29	Moderate quality. Adequate theoretical foundation, but lacks reflexivity.
Article 4	3	2	3	2	1	3	2	0	2	3	3	2	3	29	Moderate-to-high quality. Methodological rigor, but lacks active participation.
Article 5	3	2	3	2	1	3	2	0	2	3	3	2	2	28	Moderate quality. Small sample size and gaps in reflexivity.
Article 6	3	2	2	2	1	2	2	0	2	3	3	2	2	26	Moderate-to-low quality. Limited instrument and basic analysis.
Article 7	3	2	2	2	1	3	2	0	2	3	3	2	2	27	Moderate quality. Clear analysis, but the limitations are poorly discussed.
Article 8	3	2	3	3	1	3	3	1	1	3	3	2	3	31	Moderate-to-high quality. Good methods and practical application, but weak reflexivity.
Article 9	3	1	3	3	0	3	3	0	1	3	3	2	3	28	Moderate quality. Solid structure, but no discussion of limitations.
Article 10	3	1	2	2	0	3	3	0	1	3	3	2	3	26	Moderate quality. Good analysis, but lacks reflexivity and methodological critique.

## Data Availability

No new data were created or analyzed in this study. Data sharing is not applicable to this article.

## Abbreviations

The following abbreviations are used in this manuscript:

QuADS	Quality Assessment with Diverse Studies
MBI-HSS	Maslach Burnout Inventory–Human Services Survey
SOC	Sense of Coherence
OSF	Open Science Framework
PRISMA	Preferred Reporting Items for Systematic Reviews and Meta-analysis
MeSH	Medical Subject Headings
JSS	Job Satisfaction Survey
IPWBW	Index of Psychological Well-Being at Work
SU	Service of Urgency
WRS	Work-related Stress
MSD	Musculoskeletal Disorder
OSI-R	Osipow Stress Inventory—Revised
